# Modulation of Autophagy–Lysosome Axis by African Swine Fever Virus and Its Encoded Protein pEP153R

**DOI:** 10.3390/cimb46100667

**Published:** 2024-10-07

**Authors:** Si-Yu Bai, Wenlian Weng, Hua Wang, Zhiying Cui, Jiajun Wu, Yajin Qu, Yuxin Hao, Peng Gao, Yongning Zhang, Lei Zhou, Xinna Ge, Xin Guo, Jun Han, Hanchun Yang

**Affiliations:** 1 Key Laboratory of Animal Epidemiology of Ministry of Agriculture and Rural Affairs, College of Veterinary Medicine, China Agricultural University, Beijing 100094, China; baisiyu1993@163.com (S.-Y.B.); wengwenlian2021@cau.edu.cn (W.W.); wanghua2464@163.com (H.W.); czy11023@163.com (Z.C.); quyajin@cau.edu.cn (Y.Q.); zhangyongning@cau.edu.cn (Y.Z.); leosj@cau.edu.cn (L.Z.); gexn@cau.edu.cn (X.G.); guoxincau@cau.edu.cn (X.G.); yanghanchun1@cau.edu.cn (H.Y.); 2 National Key Laboratory of Veterinary Public Health Safety, College of Veterinary Medicine, China Agricultural University, Beijing 100094, China; 3 China Animal Disease Control Center, Beijing 100125, China; wujiajun82@126.com (J.W.); 18810121512@163.com (Y.H.)

**Keywords:** African swine fever virus, pEP153R, autophagy, lysosomes, lysosome-associated membrane protein

## Abstract

The autophagy–lysosome axis is an evolutionarily conserved intracellular degradation pathway which constitutes an important component of host innate immunity against microbial infections. Here, we show that African swine fever virus (ASFV), one of most devastating pathogens to the worldwide swine industry, can reshape the autophagy–lysosome axis by recruiting the critical lysosome membrane proteins (LAMP1 and LAMP2) to viral factories while inhibiting autophagic induction in macrophages. The screening of viral membrane proteins led to the identification of several ASFV membrane proteins, exemplified by viral protein pEP153R, that could significantly alter the subcellular localization of LAMP1/2 when expressed alone in transfected cells. Further analysis showed that pEP153R was also a component of viral factories and could induce endoplasmic reticulum (ER) retention of LAMP1/2, leading to the inhibition of the fusion of autophagosomes with lysosomes. Interestingly, the ASFV mutant lacking *EP153R* could still actively recruit LAMP into viral factories (VFs) and inhibit autophagic flux, indicating the existence of a functional redundancy of other viral proteins in the absence of pEP153R and highlighting the complexity of ASFV replication biology. Taken together, our results reveal novel information about the interplay of ASFV with the autophagy–lysosome axis and a previously unrecognized function of ASFV protein pEP153R in regulating the cellular autophagic process.

## 1. Introduction

Over the last 100 years, African swine fever (ASF) has remained the most devastating threat to the worldwide swine production system due to its highly contagious, lethal nature that affects both domestic and wild pigs [1]. The causative agent, African swine fever virus (ASFV), was first described in 1921 in Kenya that reported the viral transmission from wild African wild suids to domestic pigs [2]. Since the late 1950s, ASFV left Africa on three occasions and spread to Europe and South America [1,3]. Most recently, it spread to China for the first time and disseminated quickly within the country, leading to devastating consequences to Chinese pork production [4,5]. At present, many countries in Africa, Europe, and Asia maintain endemic status with ASFV [6,7], but unfortunately, there are no effective vaccines or anti-viral drugs yet available. Phylogenetically, ASFV is a nucleocytoplasmic, large DNA virus and the only member of the *Asfarviridae* family. It displays an icosahedral morphology with an average diameter of 200 nm and contains five structural layers, including the outer envelope, capsid, inner envelope, core shell, and nucleoid [8]. Significantly, it has a large genomic size (170~200 kb) and harbors more than 150 open reading frames (ORFs), with over 50 encoded proteins found within the virions [9,10,11], while the biological functions of most of the encoded proteins have remained poorly characterized, hindering a comprehensive understanding of the complex biology of ASFV.

To make a productive life cycle in a susceptible cell such as macrophages, ASFV has to counteract or disarm host innate immunity [1,12]. While most efforts are directed toward the interaction with cellular interferon signaling [13,14], another line of defense is also essential, that is, the autophagy–lysosome axis—an intracellular sensing and degradation pathway that normally functions to maintain cellular homeostasis and to help the cell weather through difficult stress conditions, such as starvation, by clearing aggregated proteins and aged, damaged, or unwanted cellular organelles [15,16]. Mechanistically, it employs selective or non-selective mechanisms to sense and target a wide range of cellular substrates for lysosomal degradation [17]. The initiation of autophagy starts with the induction of a double-membrane structure known as the phagophore that can originate from various cellular membrane compartments, including the endoplasmic reticulum (ER), endosomes, Golgi complexes, and plasma membranes [18,19,20]. The years of research has increasingly shown that autophagy is an integral part of cellular immunity and plays a key role in sensing invading viral pathogens, inducing degradation, activating I-IFNs via pattern recognition receptors [21,22], or modulating the cellular inflammatory responses [21,23,24], leading to the activation of adaptive immunity [25,26,27].

Many viruses have evolved various strategies to modulate cellular autophagy or the autophagic process for virus replication, assembly, and release [28,29,30,31], including herpesviruses [32], hepatitis C virus (HCV) [33], hepatitis B virus (HBV) [34], influenza virus [35], pseudorabies virus (PRV) [36], porcine reproductive and respiratory syndrome virus (PRRSV) [37], and so on. In the case of ASFV, a previous study showed that the Vero-adapted ASFV strain (BA71v) could inhibit autophagy in cultured cells by activating Akt and the mammalian target of rapamycin (mTOR) complex 1 that switches off autophagy [38], but the mechanisms of regulation remain unclear. In this report, we started with the assessment of the infection by a type II virulent strain HN09 on the autophagy–lysosome axis, and then the screening of viral proteins involved in the regulation of this pathway. We found that ASFV infection could inhibit autophagic flux and result in the relocation to viral factories of lysosome-associated membrane protein1 (LAMP1) and lysosome-associated membrane protein2 (LAMP2), the major lysosome membrane proteins and the key regulators in the fusion of autophagosomes with lysosomes [39,40]. In addition, we identified several key proteins, including viral protein pEP153R, that could impair the trafficking of LAMP1 and LAMP2 to lysosomes, resulting in the inhibition of autophagosome–lysosome fusion. The details are described below.

## 2. Materials and Methods

### 2.1. Ethics and Biosafety Statement

Primary porcine alveolar macrophages (PAMs) derived from one-month-old SPF pigs were isolated as previously described [41] in accordance with the Chinese Regulations of Laboratory Animals—The Guidelines for the Care of Laboratory Animals (Ministry of Science and Technology of People’s Republic of China) and Laboratory Animal-Requirements of Environment and Housing Facilities (GB 14925±2010, National Laboratory Animal Standardization Technical Committee). The license number was AW72903202-1-2, which was approved by the Laboratory Animal Ethical Committee of China Agricultural University. All experiments involving live ASFV were performed in the biosafety level 3 laboratory at the China Agricultural of University (license number: 2022-ASFV-002).

### 2.2. Cells and Viruses

Vero cells (African green monkey kidney epithelial cells, #ATCC CRL-1587) were grown in Dulbecco’s modified Eagle’s medium (DMEM) (12491015, Gibco, New York, NY, USA) with 10% fetal bovine serum (FBS) (16140071, Gibco, New York, NY, USA), penicillin (50 U/mL), and streptomycin (50 μg/mL) at 37 °C under a humidified atmosphere of 5% CO_2_. PAMs and 3D4/21 cells (Porcine alveolar macrophage cell line, #ATCC CRL-2843) were maintained in RPMI-1640 (#61870044, Gibco, New York, NY, USA) medium, containing 10% FBS, penicillin (50 U/mL), and streptomycin (50 μg/mL) at 37 °C in a humidified atmosphere of 5% CO_2_. Genotype II ASFV strain CADC_HN09 (ASFV-HN09) (GenBank accession no: MZ614662.1) was used throughout this study. ASFV-HN09 and the recombinant viruses were all propagated in PAMs.

For virus growth kinetics, PAMs cultured in 12-well plates were infected with ASFV-WT or ASFV-ΔEP153R at an MOI of 0.1. After 1 h of incubation at 37 °C, PAMs were washed three times with RPMI-1640 medium and maintained in RPMI-1640 medium containing 2% FBS and penicillin (50 U/mL) and streptomycin (50 μg/mL). At the different time points post infection, the medium and cells were harvested and freeze–thawed before virus titration using an endpoint dilution assay on PAMs. The cells were stained with a monoclonal antibody to p30. The virus titer is expressed as 50% of the tissue culture infective dose (TCID_50_) calculated by the Reed–Muench method.

### 2.3. Antibodies and Reagents

Rabbit anti-LC3 polyclonal antibody (pAb) (ab192890, Abcam, Cambridge, UK) and rabbit anti-SQSTM1 polyclonal antibody (pAb) (ab91526, Abcam, Cambridge, UK) were purchased from Abcam. Mouse anti-Flag monoclonal antibody (mAb) (F1804, Sigma, St. Louis, MO, USA) was purchased from Sigma. Mouse anti-LAMP1 mAb (MCA2315GA, Bio-rad, Hercules, CA, USA) was obtained from Bio-rad. Rabbit anti-LAMP2 pAb (7823-1-AP, Proteintech, Wuhan, China) was obtained from Proteintech. Mouse anti-pEP153R, mouse anti-p30, rabbit anti-p54, and rabbit anti-Myc were prepared in our laboratory. Horseradish peroxidase (HRP)-conjugated goat anti-mouse IgG or anti-rabbit IgG were purchased from ZSGB-Bio. Alexa Fluor 488-conjugated goat anti-rabbit or mouse IgG (H+L) F(ab’)2 fragment, Alexa Fluor 568-conjugated goat anti-rabbit or mouse IgG (H+L) F(ab’)2 fragment, and Alexa Fluor 647-conjugated goat anti-rabbit IgG (H+L) F(ab’)2 fragment were purchased from Thermo Fisher. All restriction enzymes were purchased from New England Biolabs Inc. Chloroquine (C6628, Sigma) was purchased from Sigma Aldrich. Rapamycin (HY-19312, MCE, Shanghai, China) and Bafilomycin A1 (HY-100558, MCE, Shanghai, China) were purchased from Med Chem Express. LysoSensor™ Red/Green DND-189 (L8010, Solarbio, Beijing, China) was obtained from Solarbio.

### 2.4. Plasmid Construction and Transfection

The gene *EP153R* of ASFV strain HN09 was cloned into the pcDNA (+)3.1 or pEGFP-C1 vectors to generate plasmids pEP153R-Myc and pEP153R-GFP. Sus scrofa *LAMP1* and *LAMP2* genes derived from PAMs were cloned and inserted into the vector pCAGGS in the frame with a sequence coding for a C-terminal Flag tag to generate plasmids pLAMP1-Flag and pLAMP2-Flag. A similar approach was used to make plasmid pmCherry-LAMP1 in which the tag mCheery was placed at the N terminus of LAMP1. Deletion mutagenesis was performed to generate pEP153R deletion mutants lacking either the transmembrane domain (TM) or the cytoplasmic tail based on the plasmid pEP153R-Myc by using the Quik Change Site-Directed Mutagenesis Kit (200523, Agilent Technologies, Palo Alto, CA, USA), leading to the generation of plasmids pEP153RΔTM-Myc and pEP153RΔCTLD-Myc, respectively. All the molecular cloning was carried out via homologous recombination using the ClonExpress II one-step cloning kit (C115-01, Vazyme, Nanjing, China). The resulting constructs were further confirmed by sequencing analysis. For transfection assays, 3D4/21 and Vero cells grown on coverslips in 12-well plates were used with the Lipofectamine 2000 DNA transfection reagent (#1168019, Invitrogen, Carlsbad, CA, USA) according to the manufacturer’s instructions.

### 2.5. Confocal Microscopy

For the immunofluorescence assay (IFA), the transfected or infected cells grown on coverslips were fixed with 4% paraformaldehyde for 10 min at room temperature (RT), washed with phosphate-buffered saline (PBS) three times for 5 min each time, permeabilized for 10 min with PBS containing 0.1% Triton X-100 and 2% bovine serum albumin (BSA), and blocked with 2% BSA-PBS for 30 min at RT. The cells were then incubated with the indicated primary antibodies [Flag mAb (1:1000), Myc pAb (1:2000), LAMP1 mAb (1:100), LAMP2 pAb (1:100), p30 mAb (1:2000), EP153R mAb (1:500), and p54 pAb (1:1000)] at 4 °C overnight and washed with PBS three times (5 min for each wash). Afterwards, the cells were incubated with the appropriate secondary antibodies at a dilution of 1:1000 for another 60 min and nuclear DNA was stained with 4′,6-diamidino-2-phenylindole (DAPI) (#62248, Thermo Fisher, Waltham, USA) for 5 min and then washed with PBS three times (5 min for each wash) and once with ddH_2_O. Finally, the coverslips were mounted on glass slides. The images were captured under a Nikon A1 confocal microscope (v. 5.21.00) and processed using Image J (V1.8.0.112).

For live cell staining, PAMs or 3D4/21 cells grown on the coverslip-bottomed dishes (#150680, Thermo Fisher, Waltham, USA) were infected with ASFV-GFP or treated with CQ (100 ng/mL, 6 h), Rapa (100 ng/mL, 12 h), or Baf-A1 (5 μg/mL, 12 h) to stimulate or inhibit cell autophagy. After 24 h of infection or transfection, the cells were washed twice with RPMI-1640 and then incubated with Lysotracker-Red/Green (#L8010, Solarbio, Beijing, China) at a dilution of 1 to 8000 for 30 min, and then washed twice with RPMI-1640 again before being observed by an inverted microscope.

### 2.6. Western Blot

The indicated cells were lysed on ice with RIPA lysate buffer (#P0013C, Beyotime, Beijing, China) supplemented with a protease inhibitor cocktail (#P8340, Sigma St. Louis, MO, USA). The amounts of proteins were quantified by using the Pierce™ BCA Protein Assay Kit (23225, Thermo Fisher, Waltham, MA, USA), and the whole lysate per sample containing 20 μg was subject to Western blot analysis. Briefly, the protein samples were separated by SDS-PAGE, transferred onto polyvinylidene difluoride (PVDF) membranes, blocked with 5% milk for 2 h, and then probed with appropriate primary antibodies at 4 °C overnight. The membranes were then washed with PBST three times (10 min for each wash) and incubated with appropriate HRP-conjugated secondary antibodies at a dilution ratio of 1:5000. Subsequently, the membranes were again washed and developed using enhanced chemiluminescence (ECL) detection reagents (Vigorous, Beijing, China) and the signals were detected with the ChemiDoc MP Imaging System (Bio-Rad, USA Hercules, CA, USA). Image J was utilized for quantifying the relative density of the loaded control and indicated protein, as well as for determining the standardized relative density by dividing the target protein relative density by that of the loaded control.

### 2.7. Generation of EP153R Gene-Deletion Mutant

Briefly, the recombinant virus was generated via homologous recombination between the ASFV-HN09 genome and the donor vector through infection and transfection procedures. The donor vector pEASY-Blunt-zero-p72-EGFP-ΔEP153R was constructed to contain an EGFP reporter gene cassette under the control of the ASFV p72 promoter that was further flanked by the upstream and downstream sequences of *EP153R* with a size of 471 bp at each side. PAMs were first infected with ASFV-HN09 (MOI = 1.0) for 1 h and then transfected with the plasmid pX335 that expresses both the sgRNA targeting *EP153R* (Table S1) and nuclease Cas9, as well as the donor vector using X-tremeGENE HP DNA transfection agents (#06366236001, Roche, Basel, Switzerland) following the manufacturer’s protocol. The cells were incubated at 37 °C under 5% CO_2_ for 24 h and observed under a fluorescent microscope. The recombinant virus was selected after 4 rounds of plaque purification in PAMs based on GFP fluorescence and confirmed by PCR and genome sequencing.

### 2.8. Statistical Data Analysis

All the graphs and relevant statistical tests used in this research were generated by GraphPad Prism version 6.00 (La Jolla, CA, USA). Statistical significance between two groups was analyzed by the two-tailed unpaired Student’s *t*-test or one-way ANOVA, and asterisks indicate the statistical significance: NS, no significance; *, *p* < 0.05; **, *p* < 0.01; ***, *p* < 0.001. Error bars indicate means ± standard deviations (SD).

## 3. Result

### 3.1. Redistribution of LAMP1 and LAMP2 to Viral Factory in ASFV-Infected Cells

**Figure 1:**
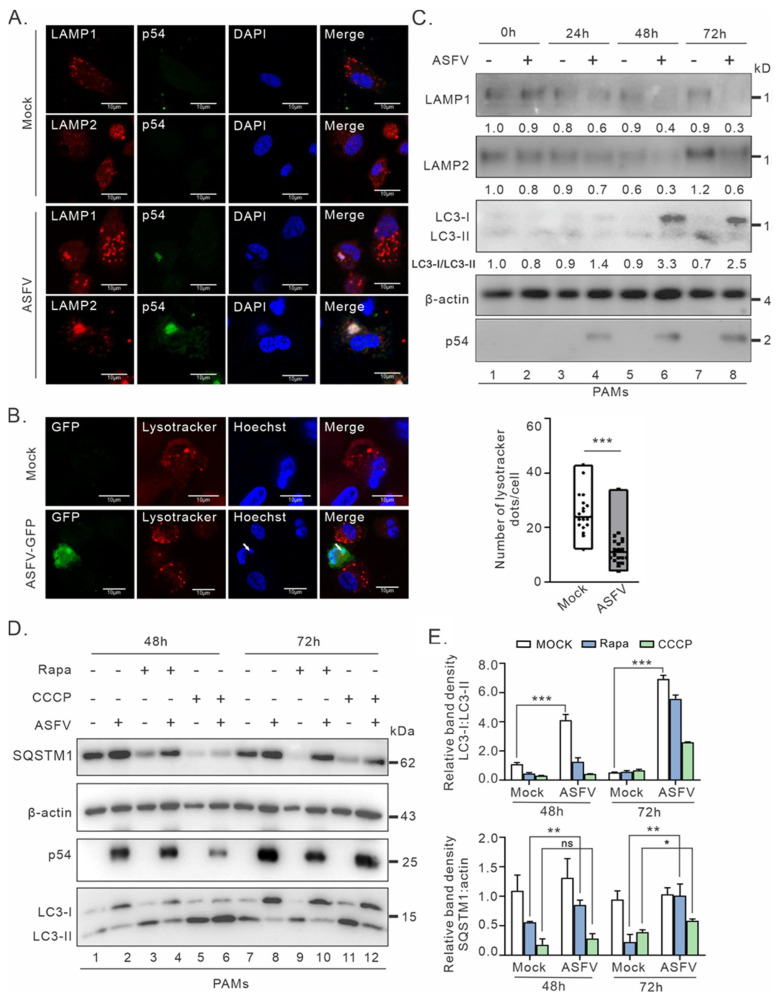
ASFV induces a redistribution of LAMP1/2 to viral factories and inhibits the induction of autophagy in infected macrophages. (**A**) Localization analysis of LAMP1/2 in ASFV-infected PAMs at an MOI of 0.5 at 24 hpi. The cells were stained with antibodies to LAMP1, LAMP2, and p54. (**B**) Effect of ASFV infection on lysosome formation. PAMs were infected with ASFV-GFP at an MOI of 0.5, and the live cells were stained with Lysotracker-Red and Hoechst at 24 hpi. (**C**) Effect of ASFV infection on the expression of LAMP1/2 and LC3-I/LC3-II. PAMs were infected with ASFV-HN09 at an MOI of 0.1 and harvested for Western blot analysis at indicated time points. β-actin was utilized as a loading control, and the viral protein p54 was employed as an indicator of infection. (**D**) Effect of ASFV infection on Rapa- or CCCP-induced autophagy. PAMs were infected with ASFV-HN09 at an MOI of 0.1. At 24 hpi, the cells were treated with Rapa or CCCP for 12 h prior to collection for Western blot analyses. β-actin was utilized as a loading control, and the viral protein p54 was employed as indicators of infection. (**E**) Quantitative analysis of LC3-I/LC3-II and SQSTM1 protein levels. The graph shows the level of LC3-I/LC3-II and SQSTM1 normalized against β-actin. The results represent at least three independent experiments. Error bars indicate standard deviations (SDs). Statistical analyses were performed by one-way ANOVA, and asterisks (*) indicate the statistical significance: ns, no significance; * *p* < 0.05; ** *p* < 0.01; *** *p* < 0.001.

To investigate how ASFV modulates the autophagy–lysosome axis, we used ASFV genotype II strain HN09 as a model organism and started with investigating the effect of viral infection on the localization of key lysosomal membrane proteins (LAMP1 and LAMP2) that are important for maintaining the function of the lysosome and the fusion of the autophagosome and lysosome. To this end, PAMs were infected at an MOI of 0.5, and at 24 h post infection, the cells were fixed for IFA analysis with antibodies to LAMP1 and LAMP2 and to p54, a viral envelope protein that is known to be a component of ASFV-induced viral factories [42]. In mock-infected cells, both LAMP1 and LAMP2 were displayed as discrete puncta in the cytoplasm as previously documented, whereas in infected cells, they exhibited an aberrant subcellular distribution and became concentrated and were colocalized with viral structural protein p54 and a DAPI-stained positive signal (ASFV nucleic acid) near the perinuclear region (Figure 1A and Figure S2), suggesting that these proteins are actively recruited to viral factories during ASFV infection.

To further confirm the alteration of LAMPs, we performed live cell staining by Lysotracker-Red, a cell acidic dye, and by Hoechst to visualize viral factories. PAMs were infected with ASFV-GFP, an ASFV derivative that is tagged with GFP at loci encoding MGF360-18R under the control of the p72 promoter and that has been reported previously [41]. The results showed that there was no apparent colocalization relationship between viral factories (Hoechst-stained ASFV-DNA) and Lysotracker. Moreover, the number and size of lysosomes (puncta of Lysotracker) were significantly reduced in ASFV-infected PAMs (Figure 1B). Thus, these results indicate that ASFV actively recruits LAMPs into viral factories. Considering that the deficiency of LAMP in lysosomes can lead to the dysregulation of the autophagic process and the accumulation of autophagosomes, we further investigated the impact on LAMP expression and autophagy flux in ASFV-infected macrophages. We found that there was a slight decrease in abundance of LAMP1 and LAMP2 at the protein level, while the accumulation of LC3-I was significantly increased (Figure 1C, lanes 6 and 8) accompanied by apparent defects in the conversion of LC3-I to LC3-II, which is consistent with a previous report [38]. Thus, the ASFV infection has a dual effect on the autophagy–lysosome axis, namely LAMP location and LC3 lipidation.

We further tested the inhibitory effect of ASFV infection on the induction of autophagy by two autophagic activators, namely carbonyl cyanide m-chlorophenyl hydrazone (CCCP) and rapamycin (Rapa), of which CCCP can induce mitophagy while Rapa activates autophagy by blocking the activation of mammalian target of rapamycin (mTOR) (Rapa). As expected, treatment with either CCCP or Rapa led to an induction of autophagy, as indicated by an increased accumulation of LC3-II and degradation of SQSTM1 (also called p62) in PAMs (Figure 1D, lanes 3, 5, 9, and 11). In contrast, ASFV infection inhibited the induction of autophagy and could inhibit the conversion of LC3-I to LC3-II and increased the accumulation of SQSTM1 in Rapa-treated PAMs at 48 hpi and 72 hpi (Figure 1D, lanes 4 and 10). Interestingly, ASFV did not inhibit CCCP-induced autophagic flux (Figure 1D,E, lanes 6 and 12). ASFV also inhibited the CCCP-induced conversion of LC3 and degradation of p62 to some extent (Figure 1D,E, lane 12). Thus, ASFV exhibits a strong ability to inhibit autophagy even in the presence of autophagy activators.

### 3.2. Screening of ASFV Proteins for Manipulating LAMP2

**Figure 2:**
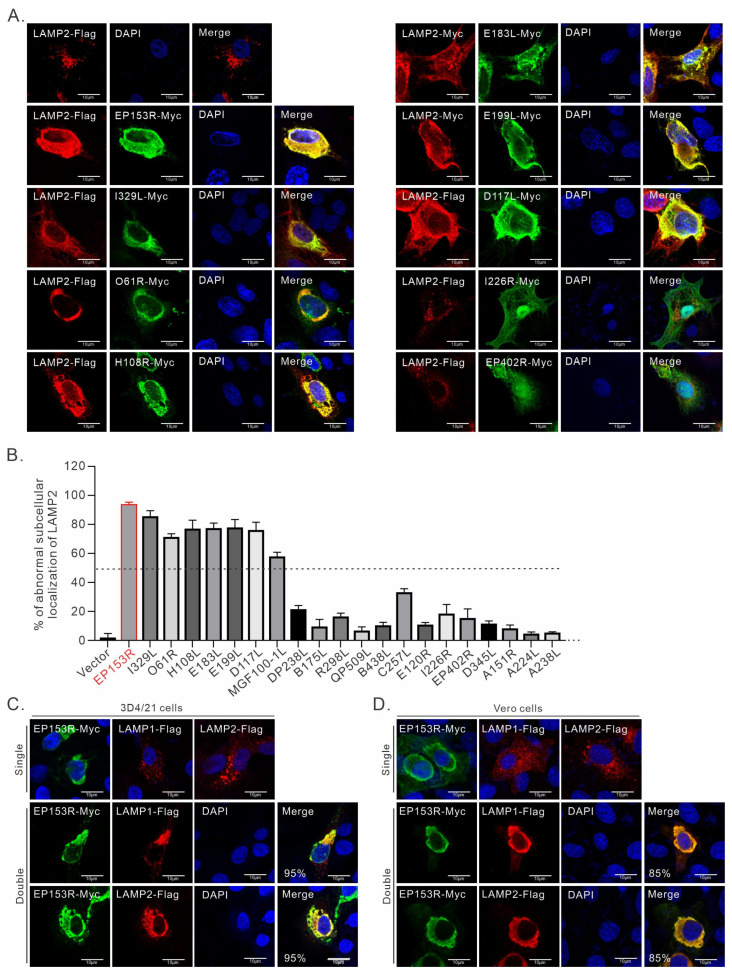
Screening of ASFV proteins for manipulating LAMP2. (**A**) 3D4/21 cells seeded on coverslips in 12-well plates were transfected to express LAMP2-Flag and different viral proteins. At 24 h post transfection, the cells were fixed and stained with antibodies to Flag (red) and Myc tags (green). (**B**) Percentage of cells expressing viral proteins colocalized with LAMP2-Flag as com-pared to co-expressed cells (*N* = 50). (**C**) 3D4/21 cells grown on coverslips in six-well plates were transfected to co-express pEP153R and LAMP1 or LAMP2. At 24 h post transfection, the cells were subject to IFA analysis. (**D**) Vero cells grown on coverslips in six-well plates were transfected to co-express pEP153R and LAMP1 or LAMP2. At 24 h post transfection, the cells were subject to IFA analysis. The proportion of cells exhibiting colocalization of LAMP1/2 and pEP153R is presented in the Merge panel. The images were captured using a Nikon A1 confocal microscope and analyzed using Image J. Oil objective: 100×. The results represent at least three independent experiments.

The fact that ASFV infection leads to the subcellular redistribution of LAMP1/2 suggests the involvement of viral membrane protein in this process. Thus, we screened viral membrane proteins necessary for LAMP1/2 re-localization. As such, we carried out a transfection assay to co-express LAMP2-Flag and a series of viral membrane proteins in 3D4/21 cells. Among the potential membrane proteins screened, seven viral proteins could induce an apparent redistribution of LAMP2, and it was clearly different from that in the singly expressed cells. Moreover, they colocalized very well in co-transfected cells (Figure 2B). These viral proteins include pEP153R, pI329L, pO61R, pH108R, pE183L, pE199L, and pD117L. Interestingly, these viral proteins are mostly inner envelope proteins. As the control, I226R-Myc and I243L-Myc did not colocalize with LAMP2. Further statistical analysis showed that pEP153R stood out as a viral protein that induced the most significant aberrant distribution of LAMP2 (Figure 2A,B), and therefore, we chose this protein for further investigation.

Retrospectively, pEP153R is a conserved transmembrane protein with a size of 153–179 amino acids that contains an extracellular domain, a transmembrane region (TM), and a C-type lectin-like domain (CTLD). Moreover, it has been shown to be implicated in viral pathogenesis [43,44]. In addition to LAMP2, we found that pEP153R could induce a redistribution of LAMP1-Flag in co-transfected 3D4/21 cells (Figure 2C). To explore whether this effect is cell-type-independent, we further conducted this experiment in Vero cells, which can be infected by ASFV and possess excellent transfection effects. The result shows that pEP153R could also induce the redistribution of LAMP1/2 in Vero cells (Figure 2D).

### 3.3. ASFV pEP153R Induces ER Retention of Both LAMP1 and LAMP2

**Figure 3:**
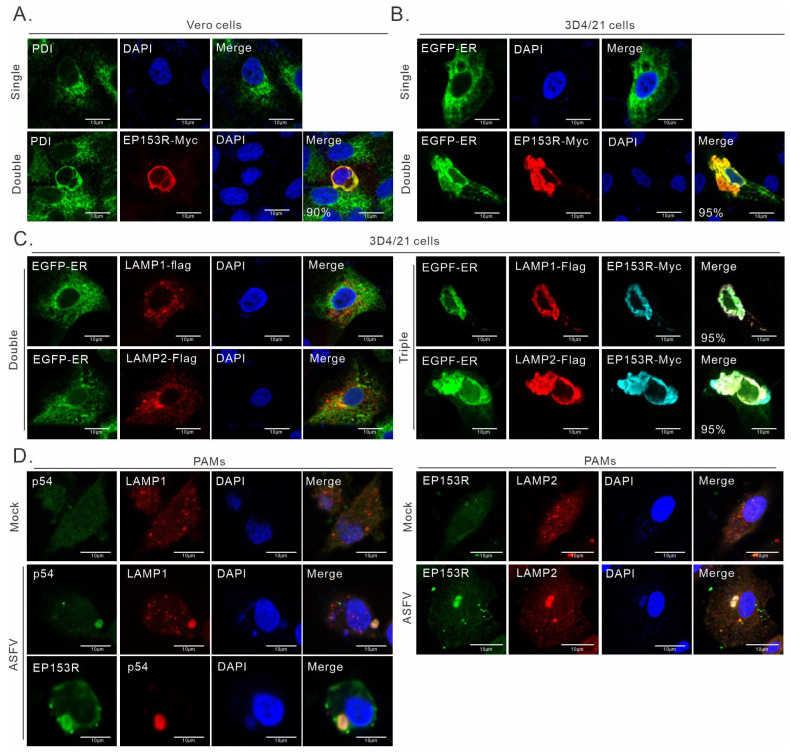
pEP153R causes ER retention of LAMP1/2 in transfected cells. 3D4/21 or Vero cells grown on coverslips in six-well plates were doubly transfected to co-express pEP153R and LAMP1, LAMP2, or ER-EGFP, or triply transfected to express pEP153R, ER-EGFP, and LAMP1 or LAMP2. At 24 h post transfection, the cells were subject to IFA analysis. ER-EGFP was used to indicate the ER. (**A**,**B**) Colocalization analysis of pEP153R with ER-EGFP, LAMP1-Flag, or LAMP2-Flag in 3D4/21 (**A**) and Vero (**B**) cells. (**C**) Effect of pEP153R on the subcellular localization of LAMP1-Flag or LAMP2-Flag in 3D4/21 cells. (**D**) Localization analysis of pEP153R with LAMP1 and LAMP2 in ASFV-infected cells. PAMs cultured on coverslips in six-well plates were infected with ASFV-HN09 (MOI = 0.5) and harvested for IFA analysis with antibodies to LAMP1, p54, LAMP2, and pEP153R. The proportion of cells exhibiting colocalization of pEP153R with different proteins is presented in the Merge panel. Oil objective: 100×. Immunofluorescence images were captured using a Nikon A1 confocal microscope and analyzed using Image J.

To further understand how pEP153R executes its function, we investigated its subcellular localization in transfected cells. The IFA analysis revealed that pEP153R is an ER resident protein that was colocalized with PDI in transfected Vero cells (Figure 3A). Similar results were observed in 3D4/21 cells (Figure 3B); however, the antibodies to PDI did not work in this cell line. Therefore, EGFP-ER, a molecule in which the ER-targeting signal (KDEL) is fused to the EGFP C-terminus, was used as a surrogate (Figure 3B). Again, pEP154R showed a good colocalization relationship with EGFP-ER. Importantly, neither LAMP1-Flag or LAMP2-Flag colocalized with ER-EGFP when co-expressed (Figure 3C, left), but in the presence of pEP153R, they became colocalized (Figure 3C, right). Thus, these results suggest that pEP153R can cause the retention of LAMP1 and LAMP2 in the ER.

We also examined the distribution of pEP153R in the condition of virus infection. It was found that both LAMP and pEP153R colocalized with p54 (encoded by E183L gene), a marker of viral factories, in ASFV-infected PAMs (Figure 3D). Although the subcellular localization pattern of pEP153R shows a huge difference between infection and transfection, LAMP1 and LAMP2 still colocalized with pEP153R gathered in viral factories (Figure 3D). Those results suggest that pEP153R probably functions as an important factor in redirecting the transport of LAMP1 and LAMP2 during infection.

**Figure 4:**
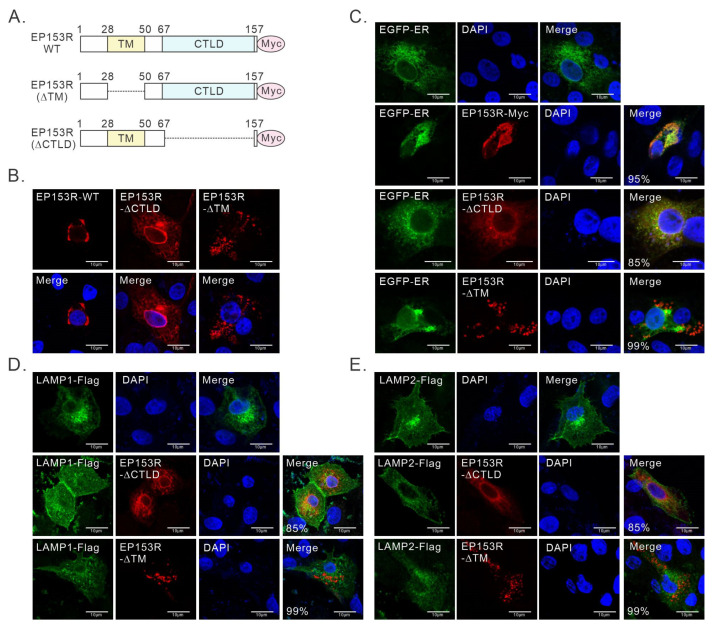
Both TM and CTLD regions of pEP153R are vital to manipulate LAMP1 and LAMP2. (**A**) Schematic representation of the construction of pEP153R mutants. (**B**–**E**) 3D4/21 cells grown on coverslips in six-well plates were transfected with the indicated plasmids to express mutants and LAMP1, LAMP2, or ER-EGFP, and were then subject to IFA analysis at 24 h post transfection. The proportion of cells exhibiting the colocalization or non-colocalization of different proteins is presented in the Merge panel. Data information: Oil objective: 100×. Images were captured using a Nikon A1 confocal microscope and analyzed using Image J.

To determine the key domain within pEP153R that causes the dislocation of LAMP1/2, we generated the deletion mutants lacking the TM (pEP153RΔTM) or CTLD (EP153RΔCTLD) (Figure 4A). The subcellular localization patterns of both pEP153R-ΔTR and pEP153R-ΔCTLDs were different from WT (Figure 4B). Moreover, the deletion failed to localize pEP153R to the ER (Figure 4C). Consequently, both mutants failed to induce a redistribution of LAMP1 or LAMP2, and the colocalization relationship was abolished (Figure 4D,E). Thus, both domains of pEP153R are necessary for LAMP1/2 redistribution.

### 3.4. pEP153R Inhibits the Colocalization of Autophagosome with LAMP1 and LAMP2

**Figure 5:**
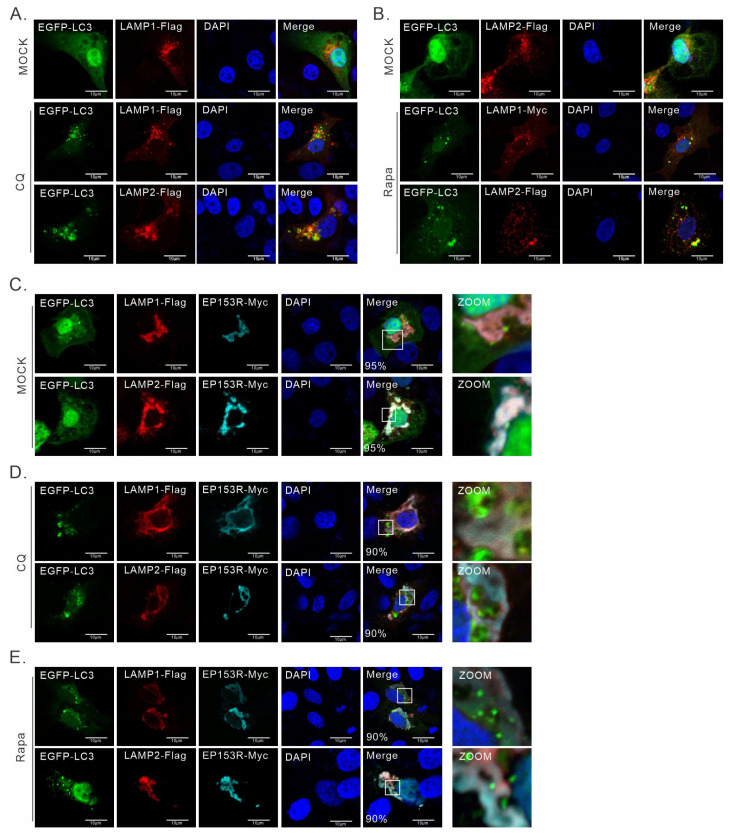
pEP153R blocks the fusion of lysosomes with autophagosomes. 3D4/21 cells grown on coverslips in six-well plates were either co-transfected to express pEGFP-LC3, pEP153R-Myc, and pLAMP1-Flag, or pLAMP2-Flag. At 24 h post transfection, the cells were treated with the indicated drugs [rapamycin (Rapa) or chloroquine (CQ)] for another 12 h, and were then subject to IFA analysis with antibodies to Flag and Myc tag. (**A**) Subcellular distribution of LC3 and LAMP1 in mock, CQ-, or Rapa-treated cells. (**B**) Subcellular distribution of LC3 and LAMP2 in mock, CQ-, or Rapa-treated cells. (**C**) Effect of pEP153R on the subcellular distribution of LC3 and LAMP2 in mock-treated 3D4/21 cells. (**D**) Effect of pEP153R on the subcellular distribution of LC3 and LAMP2 in CQ-treated 3D4/21 cells. (**E**) Effect of pEP153R on the subcellular distribution of LC3 and LAMP2 in Rapa-treated cells. The proportion of cells exhibiting a colocalization of pEP153R with different proteins is presented in the Merge panel. Data information: Oil objective: 100×. Images were captured using a Nikon A1 confocal microscope and analyzed using Image J.

LAMP, an important lysosomal membrane protein, mediates and participates in the fusion of lysosomes with autophagosomes [40,45]. To investigate whether the abnormal localization of LAMP has an effect on the fusion of autophagosomes with lysosomes, we co-expressed LAMP1-Flag or LAMP2-Flag with EGFP-LC3 and EP153R-Myc in 3D4/21 cells. In the absence of pEP153R, EGFP-LC3 exhibited a diffusive distribution throughout the cells but with most in the nucleus, whereas LAMP1 and LAMP2 were localized within lysosomal compartments (Figure 5A,B). However, in the presence of pEP153R, upon the co-expression of pEP153R with EGFP-LC3 and LAMP1 or LAMP2 in 3D4/21 cells, a few EGFP-positive autophagosomes could be observed (Figure 5C). This suggests that pEP153R does not activate autophagy, but it does exert a discernible influence on the autophagic flux.

Autophagic flux and punctate LC3 were maintained at a low level in 3D4/21 cells in normal conditions, which is not conducive to investigating the influence of pEP153R on autophagosome formation. Therefore, two autophagy regulatory drugs were selected to amplify the autophagic signals [46]. They were Rapa and chloroquine (CQ), where Rapa activates autophagy, whereas CQ can inhibit the acidification of autolysosomes and endosomes. After treatment with CQ or Rapa, we observed significant amounts of punctate aggregates of EGFP-LC3 and LAMP1/2 double-positive signals (Figure 5D,E), indicating that both LAMP1 and LAMP2 are involved in the fusion process of autophagosomes with lysosomes. However, in the presence of pEP153R, both LAMP1 and LAMP2 were wrapped within the pEP153R-positive structure (ER retention), but with no or little colocalization, suggesting that pEP153R can inhibit the fusion of autophagosomes with lysosomes in response to CQ or Rapa treatment. Those results indicate that pEP153R retains LAMP1 and LAMP2 within the ER, preventing LAMP1 and LAMP2 from participating in the formation of autolysosomes.

### 3.5. pEP153R Inhibits Fusion of Autophagosome with Lysosomes

**Figure 6:**
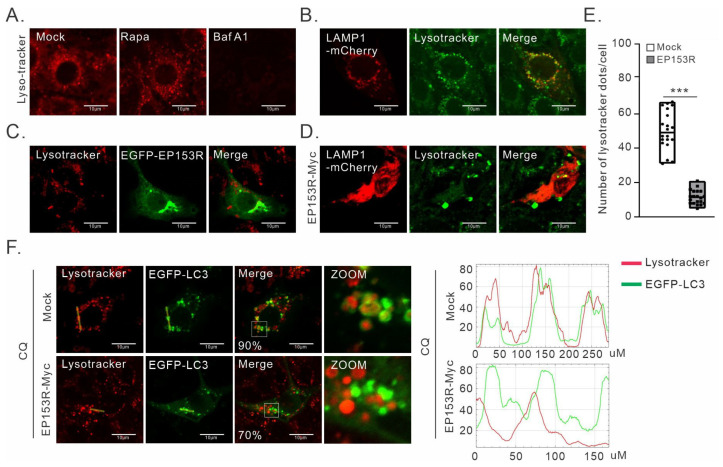
pEP153R inhibits the colocalization of autophagosomes with lysosomes. 3D4/21 cells were seeded into 35 mm dishes and transfected with the indicated plasmids. At 24 h post transfection, the cells were stained with Lysotracker-Red. (**A**) Effect of Rapa or Baf-A1 treatment on acidic vesicles stained with Lysotracker-Red. (**B**) Colocalization analysis of LAMP1-mCherry with lysosomes. (**C**) Colocalization analysis of EGFP-EP153R with lysosomes. (**D**) The effect of pEP153R on the LAMP1-mCherry localization. The abnormal localization of LAMP1 was used to indicate the cells with a co-expression of pEP153R-Myc and LAMP1-mCherry. (**E**) Quantification of the number of acidic vesicles per cell under different treatments. (**F**) Effect of pEP153R on the fusion of autophagosomes with lysosomes. 3D4/21 cells were transfected to express EGFP-LC3 or EP153R and EGFP-LC3. At 24 h post transfection, the cells were treated with CQ for 12 h before being stained with Lysotracker-Red. A statistical analysis of the colocalization between GFP-LC3 and Lysotracker was performed. Oil objective: 100×. The images were captured using a Nikon A1 confocal microscope and analyzed using Image J. Statistical analyses were performed by one-way ANOVA, and asterisks (*) indicate the statistical significance: *** *p* < 0.001. Error bars indicate standard deviations (SDs).

To further elucidate whether pEP153R can block the transport of LAMP to lysosomes, we utilized Lysotracker to label the acidic organelles. A strong signal of Lysotracker staining could be detected in Rapa-treated or untreated cells, but not in cells treated with Bafilomycin A (Baf-A1), an inhibitor of lysosome acidification [46], thus confirming the reliability of Lysotracker-Red for staining acidic organelles in 3D4/21 cells (Figure 6A). To enable the live cell visualization of LAMP1 and pEP153R in conjunction with Lysotracker, we generated plasmids expressing EGFP-tagged pEP153R and mCherry-tagged LAMP1. There was a significant colocalization relationship between Lysotracker Green and LAMP1-mCherry (Figure 6B), which unequivocally validates the utility of both LAMP1 and Lysotracker as reliable markers for late endosomes/lysosomes in normal cells.

The analysis of EGFP-EP153R and Lysotracker signals indicated that pEP153R exhibits no colocalization with lysosomes, but the over-expression of pEP153R could lead to a reduction in lysosome density (Figure 6C–E). Due to the inability to simultaneously observe the Lysotracker, LAMP, and pEP153R, the abnormal localization of LAMP1-mCherry was used to indicate the presence of pEP153R-Myc. The distribution pattern of LAMP1-mCherry resembled that of the ER structure and did not colocalize with the fluorescence of Lysotracker in pEP153R-expressed cells (Figure 6B,D). Thus, these results indicate that EP153R can inhibit the transport of LAMP1 from the ER to lysosomes. Next, we investigate whether pEP153R is also able to inhibit the fusion of autophagosomes and lysosomes. Neither EGFP-LC3 puncta colocalized with Lysotracker when co-expressed with pEP153R in CQ-treated 3D4/21 cells, but in the absence of pEP153R, they became colocalized (Figure 6F). This result shows that the fusion between autophagosomes and lysosomes were obviously inhibited by pEP153R.

### 3.6. There Is a Functional Redundancy in Viral Proteins for Manipulating LAMP and Autophagy

**Figure 7:**
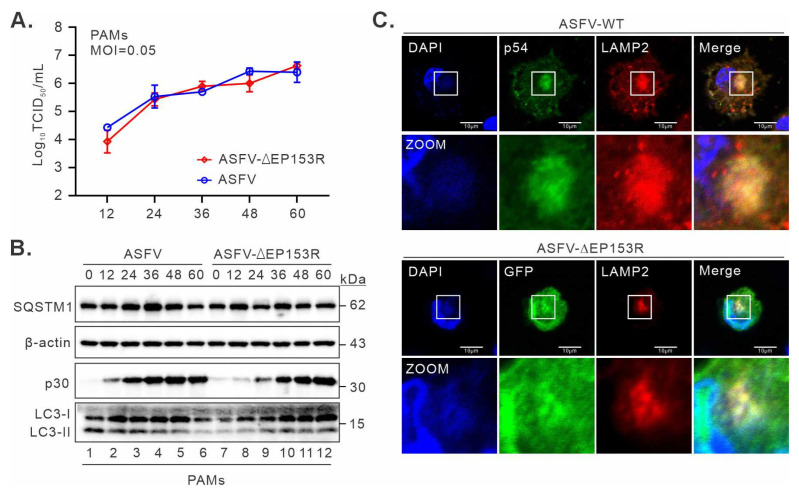
Deletion of the *EP153R* gene did not affect the replication of ASFV and its effect on autophagy and LAMP. (**A**) Growth kinetics analysis of ASFV-WT or ASFV-ΔEP153R at an MOI of 0.1. (**B**) The effect of infections by ASFV-WT or ASFV-ΔEP153R on SQSTM1 and LC3-I/LC3-II tested by Western blot with indicated antibodies. β-actin was utilized as a loading control, and the viral protein p30 was used as an indicator of infection. (**C**) Subcellular localization of LAMP1 and LAMP2 in WT or ASFV-ΔEP153R-infected cells. PAMs were cultured on coverslips in six-well plates and infected with WT or ASFV-ΔEP153R (MOI = 0.5). At 24 hpi, the cells were fixed, permeabilized, and then stained with antibodies to LAMP2 and p54. Data information: The results represent at least three independent experiments. Error bars indicate standard deviations (SDs). Oil objective: 100×. The images were acquired by a Nikon A1 confocal microscope and processed using Image J.

To further validate the role of pEP153R in the dislocation of LAMP1 and LAMP2 during ASFV infection, a recombinant ASFV lacking the EP153R gene (ASFVΔEP153R) was generated. The EP153R gene was replaced with a cassette containing the fluorescent gene EGFP under the control of the ASFV p72 promoter. The growth characteristics of ASFVΔEP153R and wild-type (WT) in vitro were first evaluated in PAMs. Interestingly, ASFVΔEP153R displayed similar growth kinetics to WT (Figure 7A), suggesting that EP153R is not essential for ASFV replication in cell culture. We also tested the effect of EP153R deletion on autophagy induction and LAMP1/2 localization. The results showed that the mutant ASFVΔEP153R retained the ability to inhibit autophagic flux with minimal discernible difference with the WT virus (Figure 7B), despite the abundance of viral protein in ASFV-ΔEP153R-infected cells being slightly lower than that in WT-infected cells (Figure 7B). In addition, LAMP remained localized in the viral factory in infected PAMs despite the absence of pEP153R (Figure 7C). Thus, these results suggest that other viral proteins likely play a redundant function in the modulation of autophagy and LAMP redistribution.

## 4. Discussion

The autophagy–lysosome axis plays a key role in eliminating protein aggregates, damaged organelles, and pathogens through lysosome degradation [19,47,48,49]. It is also a double-edged sword, as it can also be hijacked by viruses to promote their own replication [50,51,52]. Hence, studies of the interplay between autophagy and ASFV are highly relevant to our understanding of the replication and immune evasion mechanisms of this pathogen. The experiments described here revealed three salient findings: (i) ASFV infection reshapes the autophagy–lysosomal axis by recruiting LAMP1/2 into viral factories and inhibits the autophagic induction in ASFV-infected PAMs. (ii) ASFV membrane protein pEP153R can induce the ER retention of LAMP1/2 and henceforth impairs autophagosome–lysosome fusion. (iii) There is a functional redundancy among ASFV-encoded proteins in the modulation of the autophagy–lysosome axis. The relevant insights and significance are discussed below.

Many viruses have evolved various strategies to escape autophagy surveillance or even reprogram the autophagic process for viral maturation, assembly, or replication [32,48,49,53], such as MeV, PPRSV, and Newcastle disease virus [54,55,56]. Previous research indicates that ASFV is capable of targeting the PI3K/Akt/mTOR axis to inhibit autophagy [38]. Similarly, multiple viruses could regulate autophagy via the PI3K/Akt/mTOR signaling pathway, such as porcine epidemic diarrhea virus (PEDV) and porcine circovirus 2 (PCV2) [57,58]. The ASFV genome’s complexity allows it to encode various proteins, which may enable it to influence several stages of the autophagy–lysosome axis, similar to HSV and HBV [59,60,61]. During ASFV infection, viral components specifically bind dynein and migrate to perinuclear viral replication sites to form viral factories that resemble cellular aggresomes [62,63]. The formation of ASFV viral factories needs to rearrange the endomembrane and cytoskeleton, which impacts many cellular responses, such as autophagy and ER stress [63]. Consistently, our results showed that ASFV infection concentrates lysosomal membrane protein LAMP1 and LAMP2 to viral factories and inhibits autophagic flux (Figure 1A,B), which are consistent with previous findings of ASFV infection in Vero cells [38,64]. ASFV can reorganize the endomembrane system and disrupt the autophagy–lysosomal system in macrophages, thereby likely impairing the crucial functions of macrophages such as phagocytosis and the immune response. This observation may provide an explanation for the ability of ASFV to establish persistent replication within macrophages and for the clinical increase in bacterial secondary infections. Future studies may be direct in further investigating the effect of ASFV infection on macrophage function in the digestion of bacterial pathogens.

Many viral membrane proteins have been demonstrated to impair the degradation of autophagosomes by interacting with autophagy-related proteins or modulating the endosomal membrane [65,66,67]. For example, SARS-CoV-2 ORF7a, a hydrophobic transmembrane protein that is located in the ER, can block the fusion of autophagosomes with lysosomes [68]. Similarly, Epstein–Barr virus (EBV) BPLF1, a large tegument protein, can attenuate the ubiquitination of p62 and impede the interaction of LC3 with p62 [69]. As for ASFV, the protein pA179L, one of the most annotated viral proteins, was found to interact with Beclin-1 and block autophagosome formation in Vero cells [70,71]. Based on our screening results, ASFV encodes several potential regulators such as pI329l, pO61R, pH108L, pE183L, pE199L, and pD117L, and these proteins likely function in a coordinated manner with pEP153R to modulate the autophagy–lysosome axis. Most of those viral proteins either possess membrane targeting signals or have been characterized as inner membrane components of ASFV [72,73,74,75]. It is possible that ASFV reshapes the host–cell endosome system to induce viral factory formation and to promote viral replication. This hypothesis warrants rigorous investigation in the near-future.

We found that pEP153R can significantly affect autophagy and the subcellular localization of LAMP, while *EP153R* gene deletion has no effect on ASFV replication and autophagy inhibition. Previous studies have also shown that the *EP153R* gene is not essential for virus replication in cells [76], but deleting both *EP153R* and *EP402R* genes affects the virulence of ASFV. And the additional deletion of *EP153R* from BeninΔDP148RΔEP402R can also further attenuate ASFV pathogenicity [43]. Moreover, the deletion of *EP153R* alone or the co-deletion of *EP153R* and *EP402R* from ASFV-G-Δ9GL induces almost undetectable viremia levels when inoculated into domestic pigs and fails to protect them against challenge with parental virulent ASFV-Georgia [44]. These results suggest that pEP153R indeed plays a key role in viral pathogenesis, but ASFV may encode multiple synergistic proteins in order to better adapt to various conditions. In this report, it shows a significant effect on the localization of LAMP1 and LAMP2, indicating a specific mechanism by which pEP153R inhibits macrophage autophagy through causing a dislocation of LAMP1 and LAMP2. Granja et al. proposed a hypothesis that pEP153R impaired the exocytosis process to inhibit MHC-I membrane expression [77]. According to our experimental findings, pEP153R could possibly influence the presentation of MHC-I by inhibiting autophagy. Further investigation shall be implemented to definitively establish whether pEP153R or ASFV-mediated autophagic inhibition is associated with the disorder of the MHC-I pathway. However, pEP153R undertakes multiple regulatory functions in cells, even after the individual knockout of *EP153R*, and ASFV can still influence the localization of LAMP1 and LAMP2. Hence, we speculate that there are still other viral membrane proteins participating in affecting the localization of LAMPs. To further ascertain the influence of changes in LAMP localization and autophagy on ASFV replication and cellular functions, it might be necessary to conduct more research on other viral proteins involved in this process.

In summary, ASFV encodes multiple viral membrane proteins to manipulate autophagy and the subcellular localization of LAMP1 and LAMP2, while the abnormal localization of LAMP may be one of the important reasons for autophagy suppression. Among them, pEP153R is a key viral protein in blocking the transport of LAMP1 and LAMP2 to lysosomes and in impairing the fusion of autophagosomes with lysosomes. Our results suggest a complex interplay between ASFV and the autophagy–lysosome axis for promoting viral replication and potentially pathogenesis. Future studies are urgently needed to further dissect the molecular mechanisms.

## Data Availability

All study data are included in the main text.

## Supplementary Materials

The following supporting information can be downloaded at https://www.mdpi.com/article/10.3390/cimb46100667/s1, Table S1: Primers used for the recombinant virus; Table S2: All ASFV gene names for genome-wide screening assay; Figure S1: Colocalization analysis of the key ASFV proteins and LAMP2; Figure S2. ASFV induces a redistribution of LAMP1/2 to viral factories in whole-field images.

## References

[1] Netherton C.L., Connell S., Benfield C.T.O., Dixon L.K. (2019). The Genetics of Life and Death: Virus-Host Interactions Underpinning Resistance to African Swine Fever, a Viral Hemorrhagic Disease. Front. Genet..

[2] Montgomery R.E. (1921). On A Form of Swine Fever Occurring in British East Africa (Kenya Colony). J. Comp. Pathol. Ther..

[3] Cwynar P., Stojkov J., Wlazlak K. (2019). African Swine Fever Status in Europe. Viruses.

[4] Zhao D., Liu R., Zhang X., Li F., Wang J., Zhang J., Liu X., Wang L., Zhang J., Wu X. (2019). Replication and virulence in pigs of the first African swine fever virus isolated in China. Emerg. Microbes Infect..

[5] Zhou X., Li N., Luo Y., Liu Y., Miao F., Chen T., Zhang S., Cao P., Li X., Tian K. (2018). Emergence of African Swine Fever in China, 2018. Transbound. Emerg. Dis..

[6] Arias M., Jurado C., Gallardo C., Fernández-Pinero J., Sánchez-Vizcaíno J.M. (2018). Gaps in African swine fever: Analysis and priorities. Transbound. Emerg. Dis..

[7] Ståhl K., Boklund A., Podgórski T., Vergne T., Abrahantes J.C., Papanikolaou A., Zancanaro G., Mur L. (2023). Epidemiological analysis of African swine fever in the European Union during 2022. EFSA J. Eur. Food Saf. Auth..

[8] Wang N., Zhao D., Wang J., Zhang Y., Wang M., Gao Y., Li F., Wang J., Bu Z., Rao Z. (2019). Architecture of African swine fever virus and implications for viral assembly. Science.

[9] Gaudreault N.N., Madden D.W., Wilson W.C., Trujillo J.D., Richt J.A. (2020). African Swine Fever Virus: An Emerging DNA Arbovirus. Front. Vet. Sci..

[10] Yáñez R.J., Rodríguez J.M., Nogal M.L., Yuste L., Enríquez C., Rodriguez J.F., Viñuela E. (1995). Analysis of the complete nucleotide sequence of African swine fever virus. Virology.

[11] Dixon L.K., Chapman D.A., Netherton C.L., Upton C. (2013). African swine fever virus replication and genomics. Virus Res..

[12] Ramiro-Ibáñez F., Ortega A., Brun A., Escribano J.M., Alonso C. (1996). Apoptosis: A mechanism of cell killing and lymphoid organ impairment during acute African swine fever virus infection. J. Gen. Virol..

[13] Wang Y., Kang W., Yang W., Zhang J., Li D., Zheng H. (2021). Structure of African Swine Fever Virus and Associated Molecular Mechanisms Underlying Infection and Immunosuppression: A Review. Front. Immunol..

[14] Franzoni G., Pedrera M., Sánchez-Cordón P.J. (2023). African Swine Fever Virus Infection and Cytokine Response In Vivo: An Update. Viruses.

[15] Mizushima N., Komatsu M. (2011). Autophagy: Renovation of cells and tissues. Cell.

[16] Parzych K.R., Klionsky D.J. (2014). An overview of autophagy: Morphology, mechanism, and regulation. Antioxid. Redox Signal..

[17] Deretic V., Kroemer G. (2022). Autophagy in metabolism and quality control: Opposing, complementary or interlinked functions?. Autophagy.

[18] Ge L., Wilz L., Schekman R. (2015). Biogenesis of autophagosomal precursors for LC3 lipidation from the ER-Golgi intermediate compartment. Autophagy.

[19] Militello R.D., Colombo M.I. (2011). A membrane is born: Origin of the autophagosomal compartment. Curr. Mol. Med..

[20] Hu Y., Reggiori F. (2022). Molecular regulation of autophagosome formation. Biochem. Soc. Trans..

[21] Cui B., Lin H., Yu J., Yu J., Hu Z. (2019). Autophagy and the Immune Response. Adv. Exp. Med. Biol..

[22] Deretic V., Levine B. (2018). Autophagy balances inflammation in innate immunity. Autophagy.

[23] Kim K.H., Lee M.S. (2014). Autophagy–a key player in cellular and body metabolism. Nat. Rev. Endocrinol..

[24] Zhang D.L., Zhang S.W., Cheng Q.H., Wu F., Wu J.D., Zhang J., Dong J.T., Zhu H.Y., Zhang S., Wu Q.Q. (2017). Effects of peritoneal macrophage autophagy on the immune function of sepsis mice. Am. J. Clin. Exp. Immunol..

[25] Kirat D., Alahwany A.M., Arisha A.H., Abdelkhalek A., Miyasho T. (2023). Role of Macroautophagy in Mammalian Male Reproductive Physiology. Cells.

[26] Wen J.H., Li D.Y., Liang S., Yang C., Tang J.X., Liu H.F. (2022). Macrophage autophagy in macrophage polarization, chronic inflammation and organ fibrosis. Front. Immunol..

[27] Wu M.Y., Lu J.H. (2019). Autophagy and Macrophage Functions: Inflammatory Response and Phagocytosis. Cells.

[28] Alirezaei M., Flynn C.T., Wood M.R., Harkins S., Whitton J.L. (2015). Coxsackievirus can exploit LC3 in both autophagy-dependent and -independent manners in vivo. Autophagy.

[29] Bright N.A., Davis L.J., Luzio J.P. (2016). Endolysosomes Are the Principal Intracellular Sites of Acid Hydrolase Activity. Curr. Biol. CB.

[30] Caragliano E., Brune W., Bosse J.B. (2022). Herpesvirus Replication Compartments: Dynamic Biomolecular Condensates?. Viruses.

[31] Chiok K., Pokharel S.M., Mohanty I., Miller L.G., Gao S.J., Haas A.L., Tran K.C., Teng M.N., Bose S. (2022). Human Respiratory Syncytial Virus NS2 Protein Induces Autophagy by Modulating Beclin1 Protein Stabilization and ISGylation. mBio.

[32] Barnes J., Wilson D.W. (2019). Seeking Closure: How Do Herpesviruses Recruit the Cellular ESCRT Apparatus?. J. Virol..

[33] Chu J.Y.K., Ou J.J. (2021). Autophagy in HCV Replication and Protein Trafficking. Int. J. Mol. Sci..

[34] Xie M., Yang Z., Liu Y., Zheng M. (2018). The role of HBV-induced autophagy in HBV replication and HBV related-HCC. Life Sci..

[35] Zhou A., Zhang W., Dong X., Liu M., Chen H., Tang B. (2022). The battle for autophagy between host and influenza A virus. Virulence.

[36] Ming S.L., Zhang S., Wang Q., Zeng L., Zhou L.Y., Wang M.D., Ma Y.X., Han L.Q., Zhong K., Zhu H.S. (2022). Inhibition of USP14 influences alphaherpesvirus proliferation by degrading viral VP16 protein via ER stress-triggered selective autophagy. Autophagy.

[37] Li J., Zhou Y., Zhao W., Liu J., Ullah R., Fang P., Fang L., Xiao S. (2023). Porcine reproductive and respiratory syndrome virus degrades DDX10 via SQSTM1/p62-dependent selective autophagy to antagonize its antiviral activity. Autophagy.

[38] Shimmon G.L., Hui J.Y.K., Wileman T.E., Netherton C.L. (2021). Autophagy impairment by African swine fever virus. J. Gen. Virol..

[39] Wilke S., Krausze J., Büssow K. (2012). Crystal structure of the conserved domain of the DC lysosomal associated membrane protein: Implications for the lysosomal glycocalyx. BMC Biol..

[40] Eskelinen E.L. (2006). Roles of LAMP-1 and LAMP-2 in lysosome biogenesis and autophagy. Mol. Asp. Med..

[41] Gao P., Zhou L., Wu J., Weng W., Wang H., Ye M., Qu Y., Hao Y., Zhang Y., Ge X. (2023). Riding apoptotic bodies for cell-cell transmission by African swine fever virus. Proc. Natl. Acad. Sci. USA.

[42] Petrovan V., Murgia M.V., Wu P., Lowe A.D., Jia W., Rowland R.R.R. (2020). Epitope mapping of African swine fever virus (ASFV) structural protein, p54. Virus Res..

[43] Petrovan V., Rathakrishnan A., Islam M., Goatley L.C., Moffat K., Sanchez-Cordon P.J., Reis A.L., Dixon L.K. (2022). Role of African Swine Fever Virus Proteins EP153R and EP402R in Reducing Viral Persistence in Blood and Virulence in Pigs Infected with BeninΔDP148R. J. Virol..

[44] Gladue D.P., O’Donnell V., Ramirez-Medina E., Rai A., Pruitt S., Vuono E.A., Silva E., Velazquez-Salinas L., Borca M.V. (2020). Deletion of CD2-Like (CD2v) and C-Type Lectin-Like (EP153R) Genes from African Swine Fever Virus Georgia-∆9GL Abrogates Its Effectiveness as an Experimental Vaccine. Viruses.

[45] Eskelinen E.L., Illert A.L., Tanaka Y., Schwarzmann G., Blanz J., Von Figura K., Saftig P. (2002). Role of LAMP-2 in lysosome biogenesis and autophagy. Mol. Biol. Cell.

[46] Pasquier B. (2016). Autophagy inhibitors. Cell. Mol. Life Sci. CMLS.

[47] Levine B., Kroemer G. (2019). Biological Functions of Autophagy Genes: A Disease Perspective. Cell.

[48] Choi Y., Bowman J.W., Jung J.U. (2018). Autophagy during viral infection-a double-edged sword. Nat. Rev. Microbiol..

[49] Ahmad L., Mostowy S., Sancho-Shimizu V. (2018). Autophagy-Virus Interplay: From Cell Biology to Human Disease. Front. Cell Dev. Biol..

[50] Chen T., Tu S., Ding L., Jin M., Chen H., Zhou H. (2023). The role of autophagy in viral infections. J. Biomed. Sci..

[51] Wu W., Luo X., Ren M. (2021). Clearance or Hijack: Universal Interplay Mechanisms Between Viruses and Host Autophagy From Plants to Animals. Front. Cell. Infect. Microbiol..

[52] Dong X., Levine B. (2013). Autophagy and viruses: Adversaries or allies?. J. Innate Immun..

[53] Paul P., Münz C. (2016). Autophagy and Mammalian Viruses: Roles in Immune Response, Viral Replication, and Beyond. Adv. Virus Res..

[54] Claviere M., Lavedrine A., Lamiral G., Bonnet M., Verlhac P., Petkova D.S., Espert L., Duclaux-Loras R., Lucifora J., Rivoire M. (2023). Measles virus-imposed remodeling of the autophagy machinery determines the outcome of bacterial coinfection. Autophagy.

[55] Chaudhary Y., Jain J., Gaur S.K., Tembhurne P., Chandrasekar S., Dhanavelu M., Sehrawat S., Kaul R. (2023). Nucleocapsid Protein (N) of Peste des petits ruminants Virus (PPRV) Interacts with Cellular Phosphatidylinositol-3-Kinase (PI3K) Complex-I and Induces Autophagy. Viruses.

[56] Cai J., Wang S., Du H., Fan L., Yuan W., Xu Q., Ren J., Lin Q., Xiang B., Ding C. (2023). NDV-induced autophagy enhances inflammation through NLRP3/Caspase-1 inflammasomes and the p38/MAPK pathway. Vet. Res..

[57] Wei L., Zhu S., Wang J., Liu J. (2012). Activation of the phosphatidylinositol 3-kinase/Akt signaling pathway during porcine circovirus type 2 infection facilitates cell survival and viral replication. J. Virol..

[58] Lin H., Li B., Liu M., Zhou H., He K., Fan H. (2020). Nonstructural protein 6 of porcine epidemic diarrhea virus induces autophagy to promote viral replication via the PI3K/Akt/mTOR axis. Vet. Microbiol..

[59] Waisner H., Lasnier S., Suma S.M., Kalamvoki M. (2023). Effects on exocytosis by two HSV-1 mutants unable to block autophagy. J. Virol..

[60] Zheng Z., Zhao M., Shan H., Fang D., Jin Z., Tang J., Liu Z., Hong L., Liu P., Li M. (2023). Noncanonical autophagy is a new strategy to inhibit HSV-1 through STING1 activation. Autophagy.

[61] Jiang Y., Han Q., Zhao H., Zhang J. (2021). The Mechanisms of HBV-Induced Hepatocellular Carcinoma. J. Hepatocell. Carcinoma.

[62] Tanaka Y., Guhde G., Suter A., Eskelinen E.L., Hartmann D., Lüllmann-Rauch R., Janssen P.M., Blanz J., von Figura K., Saftig P. (2000). Accumulation of autophagic vacuoles and cardiomyopathy in LAMP-2-deficient mice. Nature.

[63] Wileman T. (2007). Aggresomes and pericentriolar sites of virus assembly: Cellular defense or viral design?. Annu. Rev. Microbiol..

[64] Dolata K.M., Fuchs W., Caignard G., Dupré J., Pannhorst K., Blome S., Mettenleiter T.C., Karger A. (2023). CP204L Is a Multifunctional Protein of African Swine Fever Virus That Interacts with the VPS39 Subunit of the Homotypic Fusion and Vacuole Protein Sorting Complex and Promotes Lysosome Clustering. J. Virol..

[65] Staring J., Raaben M., Brummelkamp T.R. (2018). Viral escape from endosomes and host detection at a glance. J. Cell Sci..

[66] Fang R., Jiang Q., Jia X., Jiang Z. (2023). ARMH3-mediated recruitment of PI4KB directs Golgi-to-endosome trafficking and activation of the antiviral effector STING. Immunity.

[67] Villalaín J. (2022). Envelope E protein of dengue virus and phospholipid binding to the late endosomal membrane. Biochim. Biophys. Acta. Biomembr..

[68] Jiao P., Fan W., Ma X., Lin R., Zhao Y., Li Y., Zhang H., Jia X., Bi Y., Feng X. (2023). SARS-CoV-2 nonstructural protein 6 triggers endoplasmic reticulum stress-induced autophagy to degrade STING1. Autophagy.

[69] Ylä-Anttila P., Gupta S., Masucci M.G. (2021). The Epstein-Barr virus deubiquitinase BPLF1 targets SQSTM1/p62 to inhibit selective autophagy. Autophagy.

[70] Banjara S., Shimmon G.L., Dixon L.K., Netherton C.L., Hinds M.G., Kvansakul M. (2019). Crystal Structure of African Swine Fever Virus A179L with the Autophagy Regulator Beclin. Viruses.

[71] Hernaez B., Cabezas M., Muñoz-Moreno R., Galindo I., Cuesta-Geijo M.A., Alonso C. (2013). A179L, a new viral Bcl2 homolog targeting Beclin 1 autophagy related protein. Curr. Mol. Med..

[72] Matamoros T., Alejo A., Rodríguez J.M., Hernáez B., Guerra M., Fraile-Ramos A., Andrés G. (2020). African Swine Fever Virus Protein pE199L Mediates Virus Entry by Enabling Membrane Fusion and Core Penetration. mBio.

[73] Anggy F.P., Nugroho W.S., Irianingsih S.H., Enny S., Srihanto E.A. (2023). Genetic analysis of African swine fever viruses based on E183L (p54) gene, circulating in South Sumatra and Lampung province, Indonesia. Vet. World.

[74] Xia N., Wang H., Liu X., Shao Q., Ao D., Xu Y., Jiang S., Luo J., Zhang J., Chen N. (2020). African Swine Fever Virus Structural Protein p17 Inhibits Cell Proliferation through ER Stress-ROS Mediated Cell Cycle Arrest. Viruses.

[75] Vuono E., Ramirez-Medina E., Silva E., Rai A., Pruitt S., Espinoza N., Valladares A., Velazquez-Salinas L., Gladue D.P., Borca M.V. (2022). Deletion of the H108R Gene Reduces Virulence of the Pandemic Eurasia Strain of African Swine Fever Virus with Surviving Animals Being Protected against Virulent Challenge. J. Virol..

[76] Gallardo C., Sánchez E.G., Pérez-Núñez D., Nogal M., de León P., Carrascosa Á.L., Nieto R., Soler A., Arias M.L., Revilla Y. (2018). African swine fever virus (ASFV) protection mediated by NH/P68 and NH/P68 recombinant live-attenuated viruses. Vaccine.

[77] Hurtado C., Bustos M.J., Granja A.G., de León P., Sabina P., López-Viñas E., Gómez-Puertas P., Revilla Y., Carrascosa A.L. (2011). The African swine fever virus lectin EP153R modulates the surface membrane expression of MHC class I antigens. Arch. Virol..

