# Supplementary material for: Modulation of Autophagy–Lysosome Axis by African Swine Fever Virus and Its Encoded Protein pEP153R
Source: Curr Issues Mol Biol. 2024 Oct 7;46(10):11236–54. doi: 10.3390/cimb46100667 (PMC11505880; doi:10.3390/cimb46100667)
Supplement: Supplementary file 1 [file cimb-46-00667-s001.zip › cimb-3215981-supplementary.pdf]

Supplementary Materials

Table S1. Primers used for recombinant virus.

Primer	Sequence (5′–3′)
sgRNA (EP153R)-F	CACCGTATTGCCCTAAAGATTGGGT
sgRNA (EP153R)-R	AAACACCCAATCTTTAGGGCAATAC
L-arm-F	GGTACCGGGCCCCCCC***CTCGAG***TATGTTCTTGAAAAAAAGCATAAACTTAA
L-arm-R	CCGGCGACCCTTGATTAATTTTGTGTTATATATTTTTCAACCG
p72-promoter-EGFP-F	AATTAATCAAGGGTCGCCGGAGGAAA
p72-promoter-EGFP-R	ACCACAACTAGAATGCAGTGAAAAA
R-arm-F	CACTGCATTCTAGTTGTGGTTATTTTTAATTACTTAAAATTTTTATATATAAGTTTTTG
R-arm-R	TCCCCCGGGCTGCAG***GAATTC***ACATGGTTTGGGTGGAGGACA

Table S2. All gene names for the ASFV genome screening.

**A104R**	**A118R**	**A137R**	**A151R**	**A179L**
A224L	A238L	A240L	ACD_00090	ACD_00120
ACD_00160	ACD_00190	ACD_00210	ACD_00240	ACD_01020
ACD_01760	ACD_01940	B66L	B117L	B119L
B125R	B169L	B175L	B354L	B385R
B438L	B475L	C62L	C84L	C122R
C129R	C147L	C257L	C315R	C475L
C962R	CP80R	CP530R	D117L	D129L
D205R	D250R	D339L	D345L	DP71L
DP79L	DP96R	DP238L	E66L	E111R
E120R	E146L	E165R	E183L	E184L
E199L	E248R	E423R	EP152R	EP153R
EP296R	EP364R	EP402R	EP424R	F165R
F334L	F778R	F1055L	G1340L	H108R
H124R	H171R	H233R	H240R	H359L
I7L	I8L	I9R	I73R	I196L
I215L	I226R	I243L	I267L	I329L
K78R	K205R	K421R	KP177R	L11L
L60L	L83L	M448R	MGF_100-1L	MGF_100-1R
MGF_110-1L	MGF_110-3L	MGF_110-7L	MGF_110-9L	MGF_110-11L
MGF_110-12L	MGF_110-13L	MGF_110-14L	MGF_300-1L	MGF_300-4L
MGF_360-1L	MGF_360-2L	MGF_360-3L	MGF_360-4L	MGF_360-6L
MGF_360-8L	MGF_360-9L	MGF_360-10L	MGF_360-11L	MGF_360-12L
MGF_360-13L	MGF_360-15L	MGF_360-16R	MGF_360-18R	MGF_505-1R
MGF_505-2R	MGF_505-3R	MGF_505-4R	MGF_505-5R	MGF_505-6R
MGF_505-9R	MGF_505-10R	NP419L	NP868R	O61R
O174L	Q706L	QP383R	QP509L	R298L
S183L	S273R			

Summary of ASFV genes screened. Red: strong relative genes; Blue: undetectable genes.

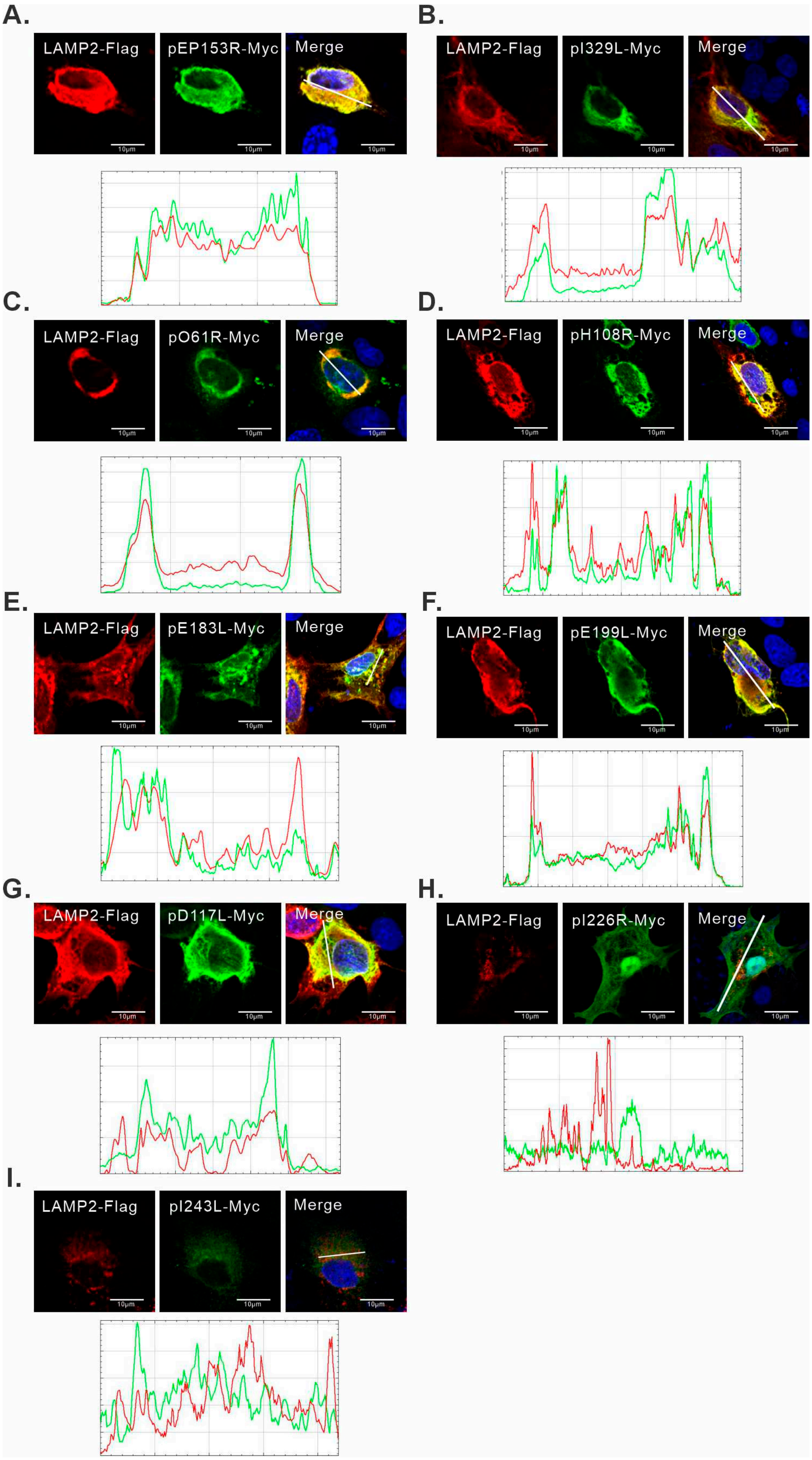

Figure S1. Co-localization analysis of the key ASFV proteins and LAMP2.

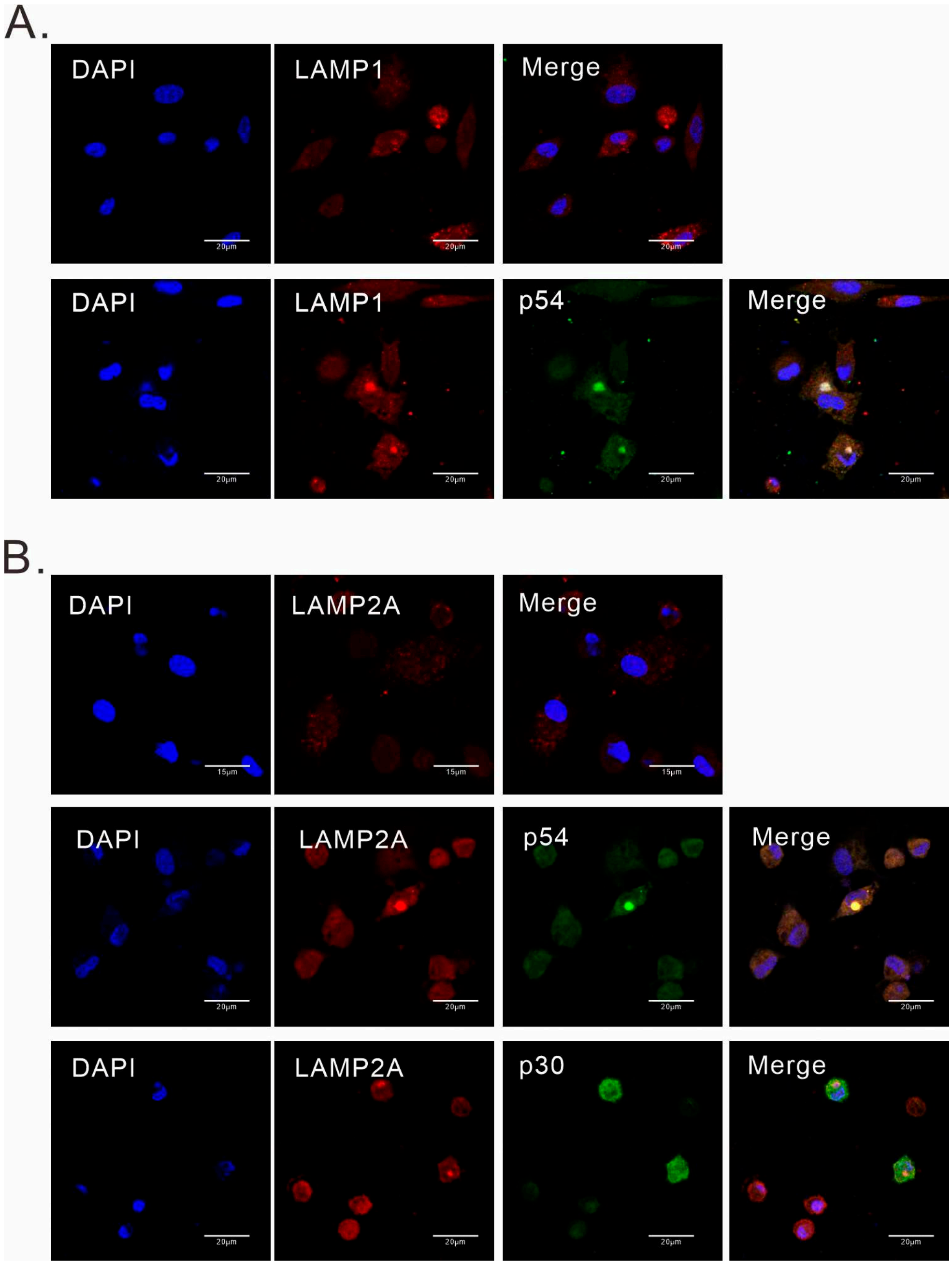

Figure S2. ASFV induces redistribution of LAMP1/2 to viral factory in whole-field images.
